# Influence of landscape on the distribution of inflammatory bowel disease in England and Wales

**DOI:** 10.1186/s12889-026-27065-1

**Published:** 2026-04-21

**Authors:** M.A Veral, R. W. Pickup, M. Roy, J. Sanderson, G. Agrawal, P. M. Atkinson

**Affiliations:** 1 Lancaster Environment Centre, Lancaster University, Bailrigg, Lancaster, LA1 4YQ UK; 2 Biomedical and Life Sciences Division, Lancaster University, Bailrigg, Lancaster, LA1 4YQ UK; 3 Department of Gastroenterology, Guy’s and St Thomas’ NHS Foundation Trust, St Thomas’ Hospital, London, UK

**Keywords:** Inflammatory bowel disease, Crohn’s Disease, Catchment, *Mycobacterium avium* subspecies *paratuberculosis*, Johne’s disease, Biogeography, Pasture

## Abstract

**Background:**

*Mycobacterium avium* subspecies *paratuberculosis* (MAP) is the bacterial pathogen which causes Johne’s disease (JD) in animals and is reported to be significantly associated with Crohn’s disease (CD) in humans. MAP is the causative bacterial pathogen of Johne’s disease in infected animals, including ruminants. Sub-clinically and clinically infected animals shed large numbers of MAP onto pastures. Previous studies have detected MAP in pasture areas deposited by both sub-clinically and clinically infected animals with Johne’s Disease and in areas outside pastures spread by non-farmed animals. Rainfall washes the pathogen from pastures via surface water and drains into rivers, thus, providing a route for human exposure from infected cattle via potable water supplies and riverine aerosols.

**Methods:**

Based on a large UK-wide IBD survey, involving 5,452 participants, we examined whether the relative risk of CD was associated with catchment-level exposure to pastures, which support a mixture of clinically and sub-clinically MAP-infected ruminants as well as disease-free animals. We used pasture proportion and hydrological transport pathways as environmental exposure indicators. Specifically, we investigated the association between the number of CD cases relative to the population (used as an offset) and biogeographical features measured at the hydrological catchment level by fitting a Poisson regression model. Covariates comprised pasture proportion (selected as a proxy for ruminant grazing), urban proportion, monthly average temperature, monthly average precipitation and river width. The same model was fitted for ulcerative colitis (UC) to provide comparative control.

**Results:**

A statistically significant relationship was found between the number of CD cases and pasture proportion, while the association between number of UC cases and pasture proportion was statistically insignificant.

**Conclusions:**

This research is the first to show an association between CD risk and the proportion of pasture at the national scale, thus, providing evidence to support the hypothesis that living within catchments with a greater proportion of pasture upon which MAP-infected animals graze may increase the likelihood of CD amongst the at-risk population.

## Introduction

Inflammatory bowel disease (IBD) encompasses conditions such as Crohn’s Disease (CD), Ulcerative Colitis (UC), Microscopic Colitis (MC) and Indeterminate Colitis (IC), all of which are characterized by chronic inflammation of the gastrointestinal (GI) tract [1–3]. It is estimated that approximately 7 million people worldwide are affected by IBD [4]. The disease is considered specific to industrially developed western countries, with the highest incidence and prevalence rates detected in North America and Europe [5]. However, a significant increase has been observed in the incidence rates of industrializing countries in the Middle East, Asia and South America [6]. IBD incidence and prevalence rates are relatively high in Europe [7–9], and in this context, the UK has one of the highest rates worldwide [7, 10].

The two major types of IBD are CD, which can affect any part of the GI tract and cause chronic inflammation of the intestine [11–13] and UC, which is limited to the colon [14–16]. CD and UC can present common symptoms, such as diarrhoea, abdominal pain and weight loss. Moreover, it is hard to determine the type of IBD in cases where symptoms are limited to the colon and no endoscopic findings are observed [17].

Although it has been emphasized that environmental, genetic and immune system regulating factors are effective in the development of IBD, it is still unclear what causes CD or UC [18]. The pathogen *Mycobacterium avium* subspecies *paratuberculosis* (MAP) is the etiologic agent of Johne’s disease (JD) in animals [19, 20], a chronic and progressive intestinal disease [21]. JD is contagious, and it can infect ruminant and non-ruminant species, including primates [22–26]. MAP is a slow-growing member of the *Mycobacterium avium* complex [23, 24, 27]. The presence of MAP in human tissues has also been found to have a significant association with CD [28–31] compared to those without IBD or ulcerative colitis [32], suggesting that MAP infection may represent an important contributing factor in disease pathogenesis, while not being sufficient on its own to cause CD [33]. Although the potential role of MAP in contributing to the onset of CD remains a topic of discussion [34–38], this has been extended to possible environmental routes through which humans might be exposed to MAP [39, 40]. A temporal lag has been reported in some countries between the importation of Johne’s diseased cattle and an increased incidence of Crohn’s Disease (e.g. Iceland, Czech Republic and Japan [41–43]).

MAP is an intracellular pathogen that can persist for long periods in an infected animal without causing clinical disease [44]. Subclinical infections have been found to occur frequently in domestic ruminants [25] and pathogenic activity has been documented globally, with notable occurrences in Europe [25, 45] and North America [46, 47]. Clinically and sub-clinically infected ruminants can shed significant numbers of MAP in their faeces onto pastures. Regardless of the intracellular persistence of MAP which causes JD, it is still shed by sub-clinically and clinically infected cattle in dung and, hence, the environment. Animals may become infectious (stage 2) after a period of time (months to years) and the shedding of MAP will increase as the disease progresses. As an animal may not exhibit (stage 3) clinical signs for many more years, undetected shedding from an infectious animal can continue for a long time [48]. MAP then persists in the environment, raising the risk of infection among a wide range of animals [49–51]. Importantly, MAP can persist outside its host for extended periods, and can be carried by surface waters into rivers and transported downstream in hydrological catchments, causing potential exposure to humans via aerosols [40, 52, 53]. We hypothesised that clusters of CD sufferers were associated with exposure to wind-driven MAP containing aerosols [39, 52]. The presence of MAP in rivers has been revealed in previous studies where MAP was detected in a significant portion of samples taken from rivers [52, 53] at concentrations of 1–10^3^ CE L^− 1^ [39]. It follows, from a hydrological perspective, that it is possible that precipitation regime and river flow have an impact on determining the distribution of MAP in the environment [52, 53].

**Fig. 1:**
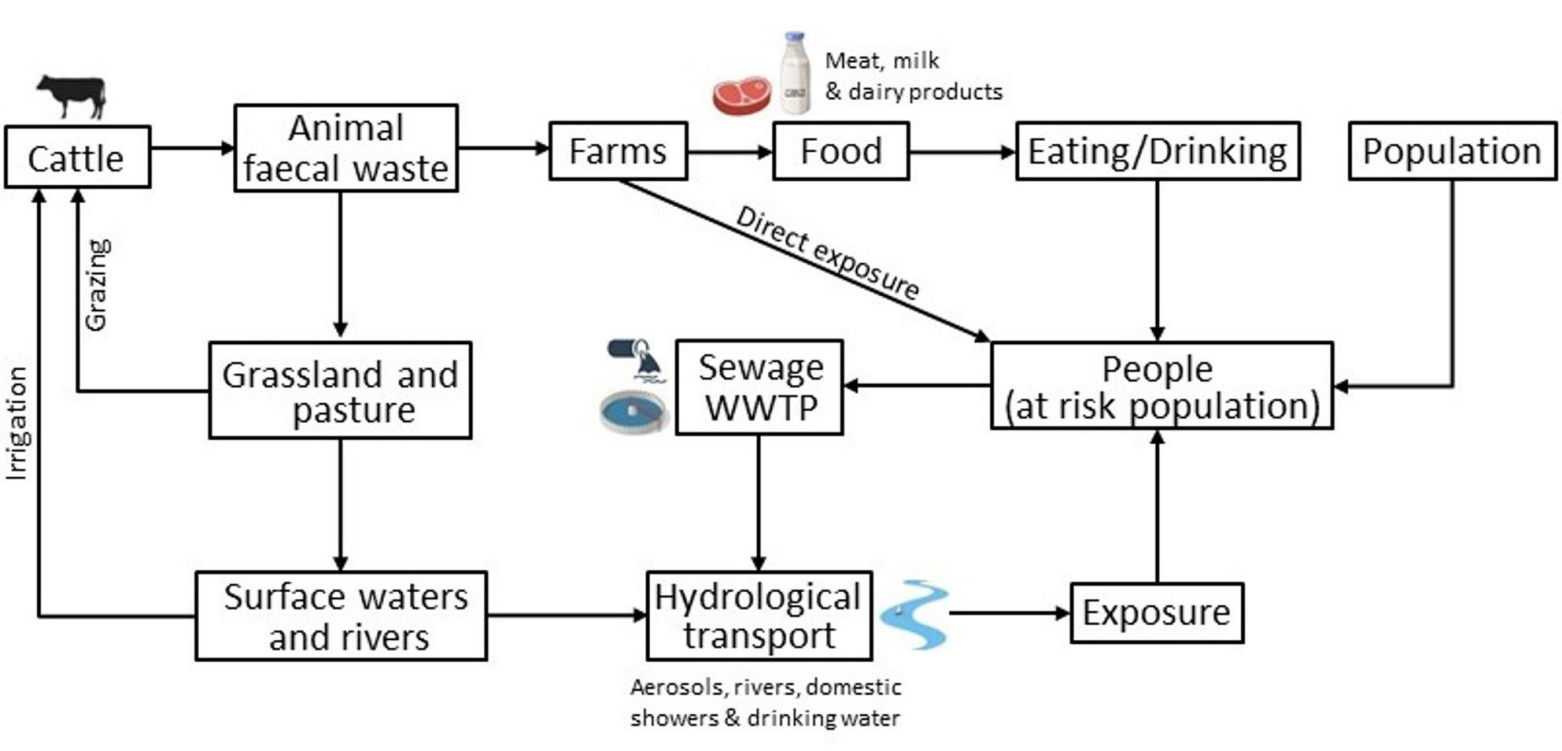
Conceptual model of transmission of MAP from infected animals to humans. (adapted from Pickup et al. 2006).

A conceptual model representing the transmission of MAP from infected animals to humans, developed initially by [53] and adapted here, is shown in Fig. 1. It is suggested that MAP enters the environment not only by infected animals, but also by farming practices such as animal slurrying practices, subsequent soil redistribution after water purification and [34, 53]. Slurrying practices are a result of shedding cattle (both dairy and beef) during winter months with their waste subsequently being spread onto the land, particularly pasture. Waste is often stored in tanks prior to spreading, but MAP survives for a prolonged period before application to the land [54]. Sheep graze on pasture all year round whilst graze cattle through spring to autumn. Further studies have detected MAP in drinking water [40, 55, 56], meat [57, 58], milk [59–63] and dairy products [64–66].

Based on the conceptual model described in Fig. 1, we framed our study to examine whether the observed distribution of the CD in the UK population is associated with biogeographical features, particularly the spatial distribution of pasture in the UK. Specifically, we used the proportion of pasture at the catchment-level as a proxy for ruminant grazing, since the association between pasture and the distribution of MAP-infected ruminants in the UK is well-established, while acknowledging that MAP itself is not directly measured in this observational study.

## Methods

### Study site and data

The study site was defined as England and Wales. A variety of data sources were used for the research including a comprehensive online survey of individuals with IBD, population and five covariates (including proportions of pasture and urban area, monthly average temperature and precipitation, and maximum river width) as well as spatial catchment boundary definitions, as detailed in this section.

### UK-wide IBD survey

A novel survey targeting individuals diagnosed with IBD across the UK was conducted, with data collected using an online platform on a purpose-built website [67] via Qualtrics (Qualtrics, Provo, UT).

The survey focused only on IBD cases living in the UK. We engaged several IBD organisations (CCUK, IBDUK, CICRA, Cure Crohn’s Colitis, Crohn’s MAP Vaccine, Guts UK and IBD Coach) to increase the survey’s profile, and ultimately response rate. During a 307-day data collection period (01 December 2021–03 October 2022), the study was featured periodically in posts from these organisations. As CCUK is the largest and most widespread IBD organisation in the UK with 47 local networks, promoting the study on its official website and social media accounts increased the likelihood of equal participation rates and even geographical coverage. Participation in the survey was entirely voluntary, and no incentives were offered. Participant selection was not target-based; access to the survey website was provided through the online channels of national IBD organizations.

Within the scope of the survey, participants were asked to share their demographic information (age, gender, ethnicity and occupation), diagnosis type (CD, UC, MC or IC), whether there was a previous IBD case in their family, and their postcodes (current, first diagnosis and previous postcodes (up to 15 years). The analysis used the postcode at first diagnosis to represent the likely area of long-term exposure, under the assumption that individuals typically reside in the same area for some time before diagnosis. Participants under the age of 16 were required to complete the survey with the consent of their guardians. Data from individuals located outside the UK and those submitting incomplete, inaccurate or unlocalized information were excluded from the final dataset used in the research. Ethical approval for the survey was obtained from Lancaster University (FHMREC20164).

### Covariates

Any measurable variable that is believed to be statistically associated with a dependent variable can be considered a potential covariate. Within the scope of this research the following covariates were selected for analysis.

- Population data and its spatial distribution across England and Wales were obtained from WorldPop with a spatial resolution of 100 m [68];
- Data on pasture and urban areas across England and Wales with a spatial resolution of 100m were obtained from the CORINE Land Cover (CLC) 2018 [69];
- Monthly average temperature (June, July and August) and precipitation (November, December and January) data between 1991 - 2020 with a spatial resolution of 2 km were obtained from the Met Office Climate Data Portal [70, 71];
- River data for England and Wales were obtained from data.gov.uk [72] and the length (m) of the widest river was used as a covariate in the regression model for each hydrological catchment area.

### Catchment boundaries in England and Wales

Figure 1 shows the conceptual environmental pathway that could represent potential indicators related to MAP originating from ruminants, including MAP from infected animals being deposited onto pasture areas and subsequently moving from pastures into rivers and surface waters downstream. To examine whether CD patterns are associated with biogeographic features connected to pasture distribution, it was decided that the analysis should be undertaken using hydrological catchment basin units as the spatial support (i.e., the map units). Consequently, humans living in each catchment are automatically and correctly associated with upstream environmental exposure indicators, such as pasture and ruminants grazing on it, within the same catchment, because both the population and these exposure indicators fall within the same spatial unit. Alternatives such as raster grids would lack this automatic association.

Data regarding the boundaries of catchment areas were obtained from The Department for Environment Food and Rural Affairs (DEFRA) for England and Wales, The Department of Agriculture Environment and Rural Affairs (DAERA) for Northern Ireland, and the Scottish Environment Protection Agency (SEPA) for Scotland. The collected catchment boundaries data were integrated into a single catchment boundaries dataset using ArcGIS Pro 3.0.

Of 495 hydrological catchment areas identified within the UK, 365 (73.73%) were in Scotland. Of the catchments in Scotland, 289 (79.17%) were found to have no survey participation. Due to the absence of survey data or very small sample sizes in Scotland, the study was conducted on hydrological catchment areas in England and Wales only.

### Poisson regression model

Poisson regression is used to model numerical data of rare events. It usually estimates the frequency of events occurring in a specific time-period or spatial area. It is particularly suitable for analysing the distribution of low-frequency events (i.e. small numbers of cases) [73] and for performing analyses based on count data, expressed as:

$$\:\mathrm{log}\left({\lambda\:}_{i}\right)={\beta\:}_{0}+{\beta\:}_{1}{X}_{1}+{\beta\:}_{2}{X}_{2}+\dots\:+{\beta\:}_{n}{X}_{n}+\mathrm{l}\mathrm{o}\mathrm{g}\left({N}_{i}\right)$$

where *λ* is the number of observed cases (i.e. number of CD or UC cases) and $\:{\beta\:}_{0}$ is the intercept. $\:{\beta\:}_{1},{\beta\:}_{2}\dots\:,{\beta\:}_{n}$ are the coefficients of covariates and $\:{X}_{1},\:{X}_{2},\dots\:,\:{X}_{n}$ are the covariates.

The model was fitted within the generalized linear model (GLM) framework using the glm function in R. Since the dependent variable (number of CD, or UC, cases participating in the IBD survey) was a count variable, the error structure was set to Poisson distribution, and the log link function was used. A log link function, which is standard for Poisson regression, was employed to model the relationship between the covariates and the expected log count of cases. The covariates used in the model (proportion of pasture and urban area, monthly average precipitation and temperature, and maximum river width) were chosen based on their theoretical relevance to environmental factors influencing disease distribution. Population$\:\left({N}_{i}\right)$, which varies across the hydrological catchment areas in England and Wales, was used as an offset term in the regression model to adjust for population differences across regions. This allows the response variable to represent proportions rather than raw counts.

Covariate selection for the final model followed an iterative approach based on the results of the initial model. Initially, all theoretically relevant covariates such as pasture and urban ratio, monthly average rainfall and temperature, and maximum river width were included in the model. After fitting the initial model, variables with large *p*-values (i.e. not statistically significant at *p* < 0.05) were identified and removed one-by-one. At each step, the model was refitted, and the impact of removing each variable on the model’s overall fit and performance was assessed. This process was repeated iteratively until only statistically significant covariates remained. The selection process ensured that the final model was both consistent and statistically valid while retaining variables with meaningful contributions to explaining the variation in the response variable.

## Results

### Exploratory data analysis

A total of 5,452 people participated in the survey. The breakdown of participants into one of four specific types of IBD was as follows: 2,672 individuals with CD (49%), 1,946 individuals with UC (35.7%), 24 individuals with MC (0.44%) and 292 individuals with IC (5.35%). 518 (9.5%) participants who provided missing, incorrect or unlocalised postcode information, resided outside the UK, did not identify their IBD type or did not provide postcode information were excluded from the final dataset. Consequently, the final adjusted distribution of cases was 2,085 for CD (53.64%), 1,559 for UC (40.10%), 19 for MC (0.48%) and 224 for IC (5.76%).

The numbers of CD and UC cases who have never changed address were found to be 182 (8.72%) and 152 (9.74%), respectively. However, the numbers of CD and UC cases who moved once or several times before diagnosis but did not move out of the catchment area were found to be 703 (33.71%) and 468 (30.01%), respectively. These findings are presented only within the scope of this exploratory analysis and were not considered in the regression model.

#### Distribution of IBD cases across England and Wales

**Fig. 2:**
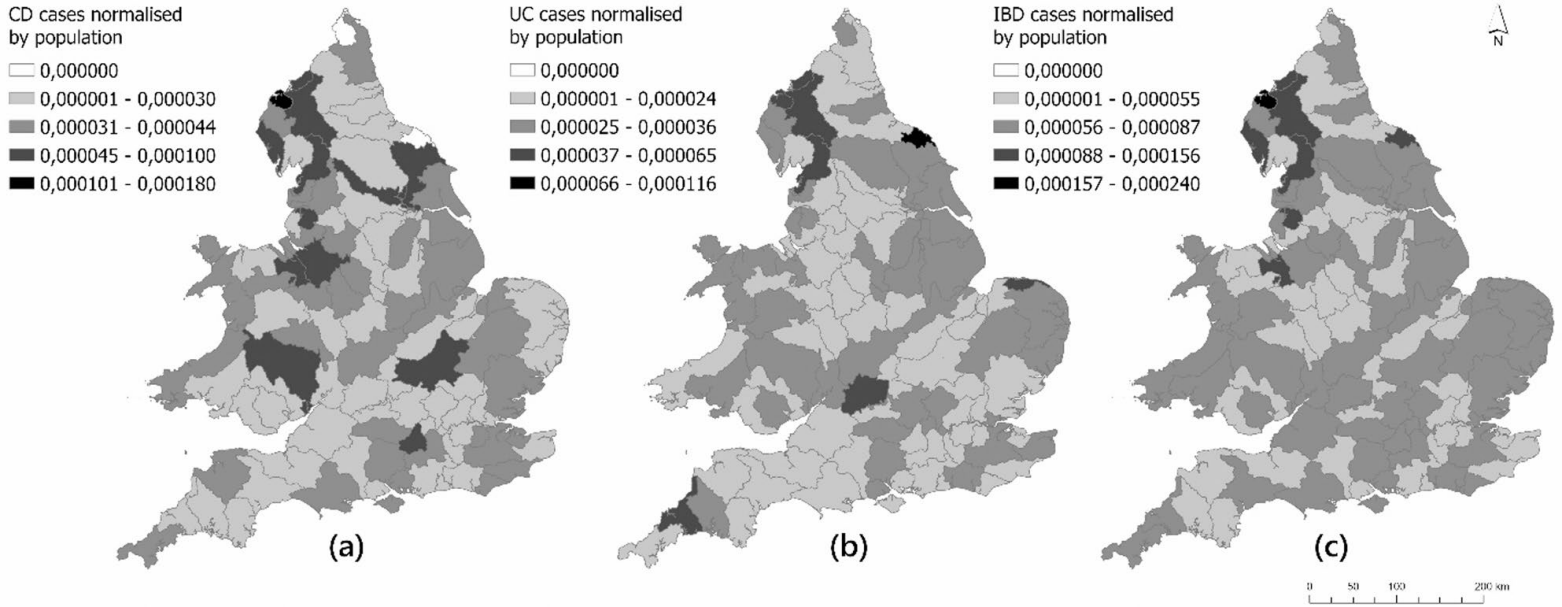
Spatial distribution of (**a**) CD, (**b**) UC and (**c**) all IBD cases in England and Wales, normalized by population. The distribution of the relative rate of participation for each disease in the survey is divided into five classes: ranging from no cases (marked in white) to the highest relative rate (marked in black). Note that since the survey response is lower than the total number of cases in the population the rate must be considered in relative terms only

First-diagnosis postcodes are crucial because the location of a patient after being diagnosed does not affect the course of the disease. CD, UC and all IBD cases in catchment areas in England and Wales were first normalized to population and then mapped (Fig. 2). Considering first the relative survey participation rate of CD cases across England and Wales, it was found that the rate of CD cases was higher in some hydrological catchment areas compared to others, although the pattern is not clear. The highest rate of UC cases relative to the population was in a catchment area near Middlesbrough, followed by some catchment areas in North-West England.

#### Distribution of covariates across England and Wales

**Fig. 3:**
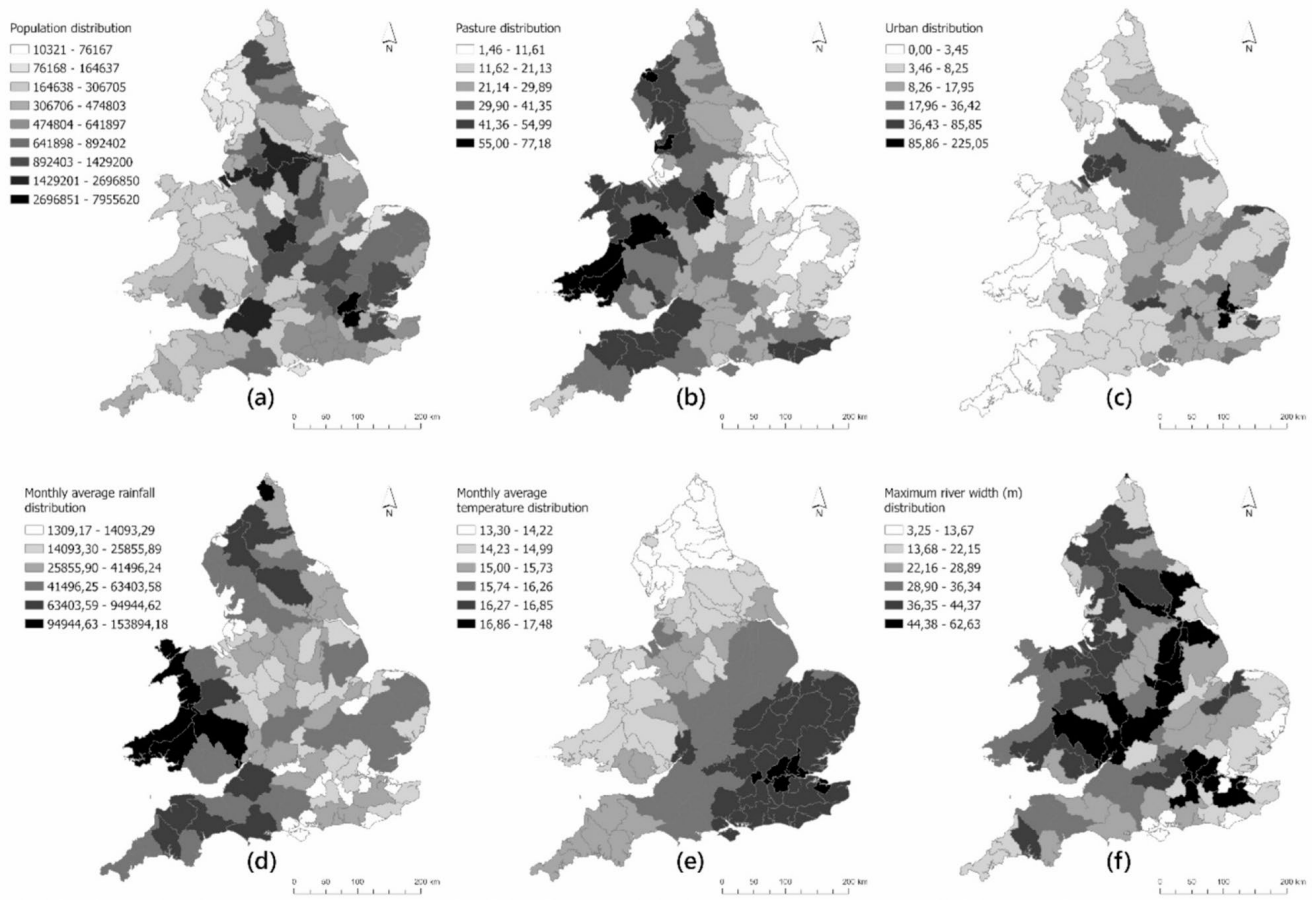
Distribution of covariates used in the Poisson regression model across England and Wales. **a** population, (**b**) pasture proportion, (**c**) urban proportion, (**d**) monthly average precipitation, (**e**) monthly average temperature and (**f**) maximum river width

Covariates considered to be related to the dependent variable ($\:\lambda\:$) were calculated for each hydrological catchment area and mapped. The distribution of these covariates across England and Wales is given in Fig. 3.

With respect to the distribution of pasture areas (Fig. 3.b), it can be observed that, as expected, the proportion increases from the eastern catchments to the western catchments. The catchments with the largest pasture proportion are located generally in the western part of the UK.

The catchments with the highest monthly average precipitation were found to be in Wales, followed by some catchment areas in the north and south-west of England (Fig. 3.d). Conversely, although some catchment areas in the south-east of England received moderate precipitation, the lowest precipitation was found in some catchment areas in the south-east (i.e. around London) and north-west (i.e. around Liverpool) of England.

Areas with the largest maximum river widths (m) were identified in regions around London and the Midlands (Fig. 3.f). Catchments with moderate to slightly above moderate maximum river widths are located in the northwest of England and in some catchments across Wales.

### Poisson regression model

The initial fitted Poisson regression model results produced a statistically significant association between CD cases and pasture proportion (*p* = 0.00724). Importantly, this relationship was not significant for UC (*p* = 0.6169; Table 1). Similarly, a statistically significant relationship was found between CD cases ​​and monthly average precipitation (*p* = 0.03046) and temperature (*p* = 0.02646), but no statistically significant relationship with urban areas and river width. Moreover, UC showed no statistical relation with any of the covariates.

**Table 1:** Results of the initial Poisson regression model established for CD and UC cases

Covariates	CD				UC			
	Estimate	Std. Error	z value	Pr (>|z|)	Estimate	Std. Error	z value	Pr (>|z|)
River width	-1.984e-03	1.815e-03	-1.093	0.27437	9.318e-04	2.115e-03	0.441	0.6596
Urban proportion	-1.737e-03	9.487e-04	-1.831	0.06715	-1.889e-03	1.174e-03	-1.608	0.1078
Pasture proportion	5.271e-03	1.963e-03	2.685	0.00724	1.160e-03	2.319e-03	0.500	0.6169
Monthly avg temp.	-6.719e-02	3.027e-02	-2.219	0.02646	-6.224e-02	3.500e-02	-1.778	0.0753
Monthly avg precip.	-2.867e-06	1.325e-06	-2.164	0.03046	1.127e-06	1.497e-06	0.753	0.4512

**Fig. 4:**
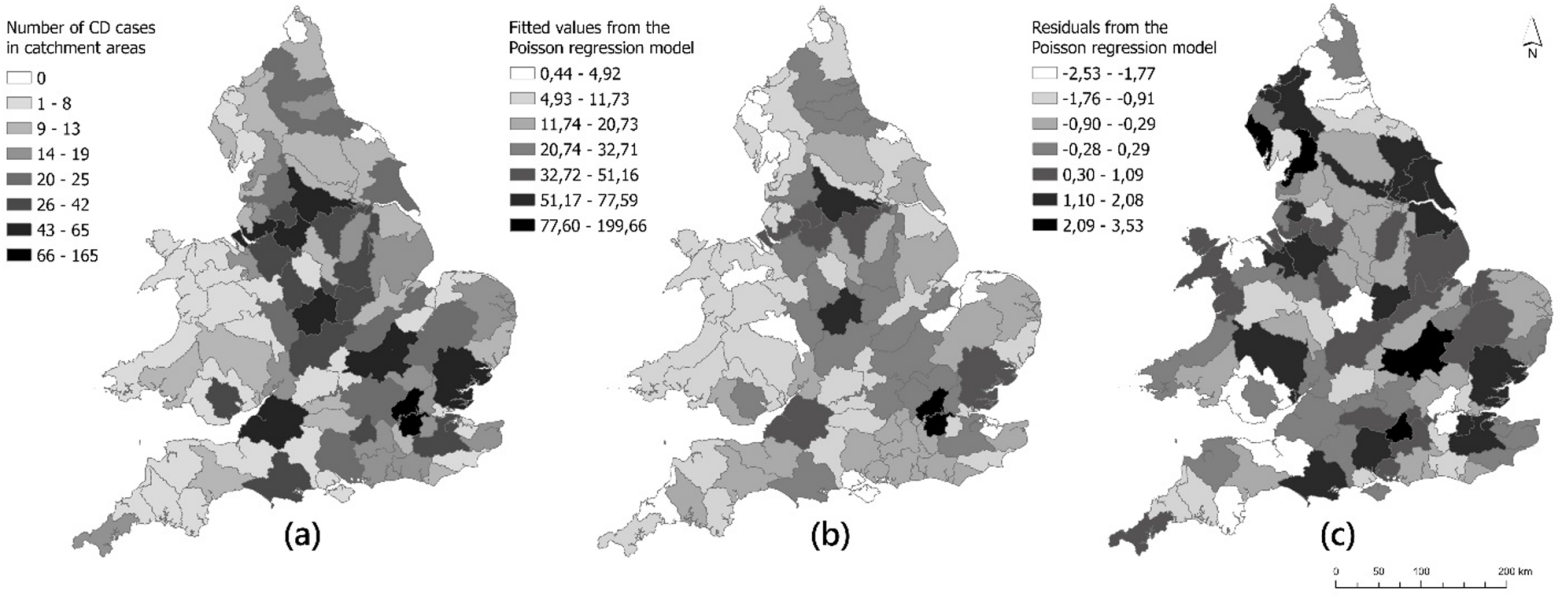
Distribution of (**a**) CD cases participating in the survey, (**b**) fitted values, and (**c**) residuals ​​predicted by the initial Poisson regression model for hydrological catchment areas in England and Wales

Considering the distribution of fitted values across England and Wales, the initial Poisson regression model predicted similarly large values ​​in some catchment areas with large numbers of CD cases who participated in the survey (i.e. some Midlands catchments and some south-eastern catchment areas of England (Fig. 4.b). Some catchment areas across England and Wales showed negative (i.e. the model over-predicted) and positive (i.e. the model under-predicted) residual values. The residual values were between − 2.53 and 3.53 and generally found to be free of large deviations with little evidence of spatial autocorrelation (Fig. 4.c).

After the initial model was fitted, the final regression model was obtained by removing the statistically non-significant parameters (𝑝 < 0.05) one-by-one for both the CD and UC models until all remaining parameters were significant, ensuring that the final set of auxiliary variables contributed significantly to explaining the variation in the response variable. In the final Poisson regression model, it was found that there was a statistically significant relationship between CD cases and pasture proportion (*p* = 6.74e-05). Differently, a statistically significant relationship was found between UC cases and monthly average temperature (*p* = 0.00126) with no association to pasture proportion.

**Fig. 5:**
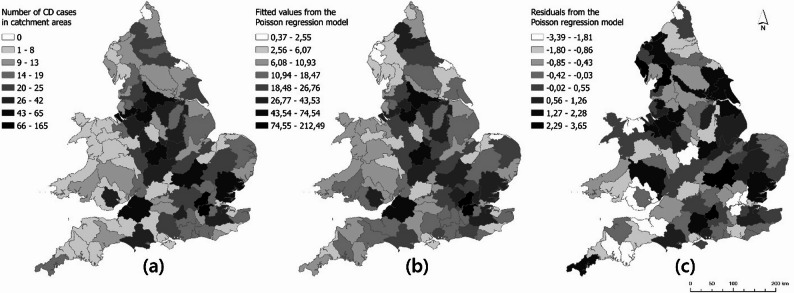
Distribution of (**a**) CD cases participating in the survey, (**b**) fitted values, and (**c**) residuals ​​predicted by the final Poisson regression model for hydrological catchment areas in England and Wales

The final Poisson regression model fitted values ​​resulted in a distribution very similar to the distribution of CD cases who participated in the survey (Fig. 5.b). Residual values ​​ranged from − 3.39 to 3.65 and showed negative and positive results as in the initial model across England and Wales (Fig. 5.c). The largest residual values ​​were found in the north of England and some Midlands catchment areas, while the lowest residual values ​​were found in catchment areas around London, in south-west England and in Wales.

## Discussion

The main question addressed in this research was whether MAP exposure is associated with CD through the hypothesized environmental pathways [40, 52, 53]. To examine this, two Poisson regression models were fitted for the number of CD cases and the number of UC cases. An initial Poisson regression model was used to inform the subsequent model refinement process. A backward stepwise elimination approach was employed to remove any statistically insignificant variables one-by-one from the initial model [74, 75]. This process aimed to ensure that only significant covariates remained in the model after covariate de-selection and, thereby, minimize model complexity. The final model presented in Table 2, which included only the pasture proportion variable, yielded a more parsimonious specification while retaining a statistically significant association between CD cases and pasture proportion (*p* = 6.74e-05). By focusing on only significant variables, the final model is simpler and more interpretable, while adequately capturing the observed association.

**Table 2:** Results of the final Poisson regression model established for CD and UC cases

Covariates	CD				UC|			
	Estimate	Std. Error	z value	Pr (>|z|)	Estimate	Std. Error	z value	Pr (>|z|)
Pasture proportion	0.006505	0.001632	3.985	6.74e-05	NA	NA	NA	NA
Monthly avg temperature	NA	NA	NA	NA	-0.08787	0.02724	-3.225	0.00126

Regarding the relationship between UC and temperature, although the final model for UC (Table 2) identified a statistically significant association with monthly average temperature (*p* = 0.00126), this does not suggest that temperature alone explains the variation in UC. Temperature variation in the UK generally follows a north-south gradient, meaning that there is a greater risk of UC in the north than in the south (since the parameter is negative), and while temperature is shown here to be associated with UC risk, other factors that vary along this gradient may also play a role. A similar argument can be made, albeit to a some extent (*p* = 6.74e-05), for the relationship between CD cases and pasture proportion. However, it is the *difference* between the CD and UC model results that is key.

The hypothesis tested in this research was whether the rate of CD cases relative to the population was related to the distribution of biogeographic features (with a specific interest in pasture areas). MAP is the etiologic agent of Johne’s disease and can affect many species (primarily sheep, cattle and goats) including primates [22, 23, 25]. Animals infected with MAP, either clinically or sub-clinically, can shed substantial amounts of the pathogen in their faeces, contaminating pastures [76]. Subsequently, MAP disseminates into the environment, where it can persist for extended periods outside a host [52, 53, 77]. Within hydrological catchments, MAP is carried to adjacent rivers through surface water flow [52, 53]. Thus, our interest was in whether a statistically significant relation could be found between the number of CD cases (while accounting for the underlying population) and biogeographic features, including pasture proportion, when analysed at the catchment level. Importantly, the relationship between the number of UC cases and biogeographic features was used as a control. By comparing the estimated parameters and their statistical significance for the CD and UC models, we were able to control effectively for other possible explanations for the significance of pasture proportion such as survey response bias related to geographical location.

The contribution of this UK-wide IBD study was, thus, to add to the limited body of knowledge on the aetiology of CD in the context of geographical factors, in addition to previous extensive studies on the relationship of IBD with environmental factors [78–81].

### Relationship to previous research

The endemic nature of Johne’s Disease worldwide is reflected in the UK [82] with an estimated JD herd prevalence of 68%, based on bulk milk samples from 225 herds [83, 84]. Following exposure to MAP, infected cattle enter a prolonged incubation period followed by the subclinical and clinical stages of infection [85]. The subclinical stage is characterized by the onset of bacterial shedding within faeces [84, 86], significantly depositing MAP onto pastures [81]. In the UK, previous studies showed that the spatial distribution of MAP increased from north to south and was significantly correlated with increasing cattle numbers over the same longitudinal axis [77], including its presence in pasture areas [76]. In this research, we found a statistically significant relationship between CD (but not UC) and pasture proportion (Table 2).

At a more local level, Pickup and co-workers showed that proximity to rivers that received water from high intensity grazing areas influenced the distribution of CD sufferers [40, 52, 53], and that aerosols, as an exposure route, may play a role in CD epidemiology [40]. Specifically, it was found that people living along stretches of the River Taff in Cardiff, Wales, UK had a greater probability of being diagnosed with CD [52]. Moreover, MAP deposition in soil from infected animals was found to be significantly associated with cattle distribution, and consequently, with pasture distribution [81]. The present study extends these findings from a national perspective. Moreover, this research, for the first time, provides evidence supporting the hypothesis that living within catchments with a greater proportion of pastureland on which MAP-infected animals graze may be associated with an increased likelihood of CD among at-risk populations, in line with the conceptual model describing the relationships between humans, animals, MAP, and the environment (Fig. 1, based on evidence from Pickup et al., 2005; 2006, and Rhodes et al., 2013). To assess the robustness of this relationship across different spatial scales, we also conducted a grid-based analysis using cells ranging from 5 km² to 50 km² (i.e. 5, 10, 15, …, 45, 50), which consistently revealed a significant positive association between pasture proportion and CD incidence. The risk of CD onset may also be associated with CD incidence, of course, with long-term exposure to other pathways such as food (beef [57], milk and dairy products [60], as well as drinking water [40, 55], but this risk is assumed to be fairly homogeneous across England and Wales at the level of the catchment units used in the analysis, and is not considered here.

Although it might be expected that farmers and veterinarians would have a higher risk of developing CD as a result of regular exposure to MAP-infected cattle, no observable increase in rates has been reported, indicating that environmental exposure alone is unlikely to be strongly associated with CD incidence [37]. Qual et al. reported a CD prevalence of 0.47% among cattle producers and veterinarians and found no association between CD and exposure to JD–infected herds [87]. In line with these studies, evidence indicating reduced IBD incidence and mortality among farmers and veterinarians relative to the general population [41, 88] does not substantiate a causal involvement of MAP in the development of CD [89]. This observation may be explained by the age-dependent nature of MAP susceptibility, the requirement for underlying host immune dysfunction, the widespread environmental presence of MAP beyond occupational settings, and the limitations of existing studies in adequately capturing early-life exposure to MAP-infected animals [37].

Importantly, the absence of elevated CD incidence among occupationally exposed adults should not be interpreted as evidence of a protective or resistant state following MAP exposure. Experimental and clinical evidence suggests that early-life exposure to MAP, particularly in genetically or immunologically susceptible hosts, is associated with an increased likelihood of subsequent clinical disease rather than the development of protective immunity [90]. As discussed by Sechi et al., MAP has been detected in intestinal tissues of both CD patients and non-affected individuals, supporting the concept that MAP may act as a contributing factor that is necessary in some individuals but not sufficient on its own for disease development [33]. Accordingly, the present findings are compatible with current biological understanding and suggest that MAP exposure alone, particularly when occurring later in life or in the absence of host susceptibility, may not translate into increased CD incidence at the population level.

### Limitations

We acknowledge the limitation that the study survey reached only a small proportion of IBD cases in the UK. According to CCUK, 540,000 people across the UK are living with Crohn’s or Colitis (CCUK, 2024). Although it was one of the largest surveys of its kind in the UK, the number of participants (3,887) accounts for approximately 1% of all IBD cases. While the sample represents only 1% of all IBD cases, it includes a wide geographic spread across England and Wales, allowing for spatial analysis. The main risk of bias lies in potential underrepresentation of certain regions, which could influence spatial patterns. However, the use of UC as a control—drawn from the same sample—mitigates against this. The differing results for CD and UC cannot be explained by possible sampling bias.

The research was conducted at the catchment level across England and Wales. This was done to capture the hypothesized conceptual links between biogeographical features such as the distribution of grazing ruminants and potential human exposure to the MAP agent. The ability to integrate the location of the potential source with the location of exposure is helpful. However, it is acknowledged that the spatial resolution of the analysis is consequently relatively coarse and, thus, while the use of UC as a control makes the evidence more compelling the results need to be treated with some caution. The results are, thus, presented as additional supporting evidence for the *possible* association between Johne’s disease/MAP [91] in ruminants and CD in humans via the MAP agent and hydrological transport pathways [52, 53]. Future research will investigate the local associations between cases and biogeographical features at a finer spatial resolution, for example, via (marked) point pattern analysis.

The case data used in this research were collected via an online survey prepared via Qualtrics and promoted at regular intervals by several voluntary IBD organisations across the UK to reach the maximum number of participants. Promotion was carried out on both the official websites and social media accounts of the organisations. Although we aimed to maximize the participation rate and create geographically equal coverage by promoting the survey widely across the UK, it was not possible to reach the entire IBD community as many sufferers may have been unaware of the survey or chose not to participate. An analysis with more participants could be helpful in revealing the spatial distribution of IBD cases across the UK with greater granularity and support the analysis proposed above. In future research, government health authorities may also be contacted to request access to the online electronic general practitioner (GP) health records database [92].

## Conclusions

In this research, the distribution of IBD cases participating in a UK-wide IBD survey, was analysed for England and Wales using a Poisson regression model to assess whether there were associations with potential biogeographic covariates. Poisson regression models were fitted for CD and UC. In the initial Poisson model, a statistically significant relationship was found between CD cases and pasture proportion (*p* = 0.00724), while no relationship was found between UC cases and pasture proportion (*p* = 0.6169; Table 1). In the final fitted model, a significant fit (*p* = 6.74e-05) was obtained between CD cases and pasture proportion (Table 2), while no such relationship was found for UC. This is the first national-scale study to report an association between CD cases and pasture proportion, suggesting that pasture areas and, thereby the distribution of ruminants, may play a role in CD risk.

## Data Availability

The datasets generated and/or analysed during the current study are not publicly available due to the presence of participants’ personal data but are available from the corresponding author on reasonable request.
